# Slide tracheoplasty for congenital tracheal stenosis with involvement of the carina and bronchi: a case report

**DOI:** 10.31744/einstein_journal/2024RC0659

**Published:** 2024-04-09

**Authors:** Fabio Eiti Nishibe Minamoto, Mariana Rodrigues Cremonese, Eduardo de Campos Werebe, Victor Nudelman, Helio Minamoto

**Affiliations:** 1 Hospital Israelita Albert Einstein São Paulo SP Brazil Hospital Israelita Albert Einstein, São Paulo, SP, Brazil.; 2 Universidade de São Paulo Faculdade de Medicina Hospital das Clínicas São Paulo SP Brazil Instituto do Coração (InCor), Hospital das Clínicas, Faculdade de Medicina, Universidade de São Paulo, São Paulo, SP, Brazil.

**Keywords:** Tracheal stenosis/congenital, Trachea/surgery, Extracorporeal membrane oxygenation, Infant, newborn, Treatment outcome

## Abstract

A female newborn presented with respiratory distress at birth and was diagnosed with congenital tracheal stenosis. The stenosis was positioned at the distal trachea and compromised the carina and the right and left bronchi. She underwent surgical treatment using circulatory life support with veno-arterial peripheral extracorporeal membrane oxygenation, and the airway was reconstructed using the slide tracheoplasty technique to build a neocarina. The patient had an excellent postoperative course, was successfully weaned from extracorporeal membrane oxygenation and invasive ventilation, and was discharged.

## INTRODUCTION

Congenital tracheal stenosis is life-threatening due to severe airway obstruction, respiratory distress, hypercapnia, and hypoxemia. This type of stenosis is a rare cause of airway stricture and a challenging diagnosis to manage. The standard treatment is airway surgical reconstruction. Close assessment of anatomical characteristics such as location, extension, involvement of the carina and bronchi, and associated cardiovascular anomalies are essential for determining the best surgical approach. Circulatory support with extracorporeal membrane oxygenation (ECMO) enables infant stabilization preoperatively and greater safety during the corrective procedures, particularly for severe and complex cases.^(1)^

The involvement of the carina and bronchi makes airway reconstruction more complex. The recommended technique in such cases is slide tracheoplasty.^(2)^ This report describes the management of a challenging case of congenital tracheal stenosis in a newborn with severe respiratory distress syndrome and a distal lesion.

## CASE REPORT

**Figure 1:**
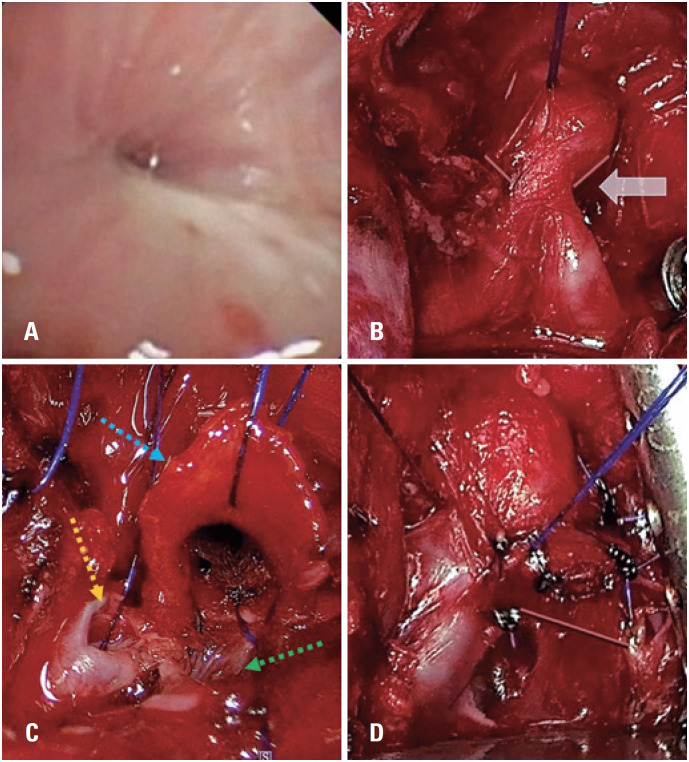
(A) Pre-operative bronchoscopy with a severe tracheal stenosis. B, C, and D: Airway reconstruction strategy; (B) Intraoperative view through mediastinal access. The white arrow indicates the stenosis where the horizontal transection was made; (C) Image after horizontal transection. Blue dotted arrow- proximal trachea; Yellow dotted arrow – right main bronchus; Green dotted arrow – left main bronchus; (D) Image after slide tracheoplasty: the anterior wall of the anastomosis was constructed from the proximal tracheal stump and the posterior wall from the distal stump

A female newborn developed signs of respiratory failure including cyanosis, agitation, gasping, and stridor. At five days of life, she underwent laparotomy at a regional hospital for surgical treatment of a presumed severe gastroesophageal reflux diagnosis. Postoperatively, she exhibited a worsened respiratory condition with refractory hypercapnia and respiratory acidosis. Computed tomography (CT) showed a distal short-segment tracheal stenosis with involvement of the carina (Figure 1A), with an extension of approximately 6mm; no other congenital anomalies were observed. The newborn was transferred to a tertiary medical facility on the tenth day of life. Following a multidisciplinary discussion, she was placed on peripheral venoarterial ECMO with cannulation of the superior vena cava and carotid artery for stabilization. Respiratory acidosis was corrected, and she was transported to the radiology department for a tomographic study that showed no associated vascular anomalies. Therefore, an airway reconstruction surgery was recommended.

She underwent flexible bronchoscopy in the operating room before airway surgery, which showed complete cartilaginous rings in the distal trachea with a severe local stricture. The procedure was performed as follows. First, a median sternotomy was performed with transpericardial access to the distal trachea and carina exposure. Next, the trachea was divided over the stenotic portions (Figure 1B).

The airway was reconstructed using the slide tracheoplasty technique with proximal and distal stumps to create the airway walls.^(2)^ This technique enlarges the tracheal lumen with a minor reduction in its extension. The proximal stump was used to build the anterior wall of the neocarina, whereas the distal stump completed the posterior wall closure (Figure 1C and D) using continuous running 5-0 PDS at both ends. After anastomosis was completed, an orotracheal tube was positioned above the anastomosis, and an air-leak test was performed. The mediastinum was drained using a 20Fr chest tube, the sternum was closed, and the patient was transferred to the intensive care unit on mechanical ventilation and ECMO support.

**Figure 2:**
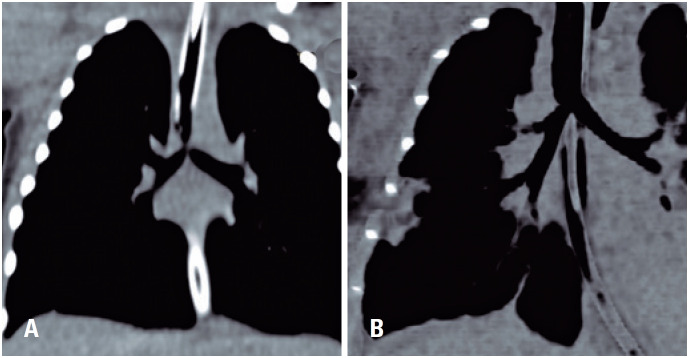
(A) Coronal reconstruction of the pre-operative chest CT with the stenosis in the distal trachea and carina; (B) Post-operative chest CT with adequate tracheal diameter

The patient was progressively weaned from ECMO after surgery and was decannulated on the first postoperative day. A control CT scan performed on the third postoperative day (Figure 2) showed no pneumomediastinum and adequate tracheal diameter. On the seventh postoperative day, the patient developed abdominal tenderness, and an abdominal radiography revealed a large pneumoperitoneum. The patient underwent exploratory laparotomy, and dehiscence of the anti-reflux repair was detected and corrected using a primary suture. On the nineteenth postoperative day, the patient was extubated and several days later was successfully discharged from the ICU. A post-operative bronchoscopy was performed which revealed that the anastomosis was widely patent and in good condition. Thus, further procedures were not required. The patient is currently in good clinical condition and is undergoing outpatient follow-up.

Histopathological examination of the resected specimen revealed a complete cartilaginous ring in the trachea and bronchi, confirming the diagnosis of congenital tracheal stenosis.

This case study was approved by the Research Ethics Committee of *Hospital Israelita Albert Einstein* (CAAE: 63999822.9.0000.0071; # 5.740.753).

## DISCUSSION

Congenital tracheal stenosis is a challenging condition which is rare and often misdiagnosed. Misdiagnosis leads to ineffective treatment and life-threatening complications. Extracorporeal membrane oxygenation support in infants is essential in cases of severe ventilatory insufficiency to ensure clinical stability, thorough investigation, and proper surgical planning. In addition, carinal and bronchial involvement increase technical difficulty, even for short-segment lesions. Slide tracheoplasty is an effective technique to repair congenital tracheal stenosis.^(3)^

In the present case, the ECMO support stabilized the patient and managed the hypoxemia and hypercapnia, allowing for the safe performance of the contrast-enhanced chest CT. This imaging modality is essential for evaluating tracheal stenosis morphology and associated cardiovascular anomalies. The images derived from the CT are critical for surgical planning, including determining the appropriate surgical approach and repair technique, and the need for extracorporeal support and concomitant heart procedures. In addition, ECMO during the procedure allows for safe and effective airway reconstruction during apnea without intermittent ventilation.

Butler et al. identified predictors of adverse outcomes in a cohort of 101 patients treated using slide tracheoplasties.^(2)^ In the multivariable analysis, bronchial stenosis, preoperative ECMO, and preoperative airway malacia yielded higher mortality rates. The current report presents two predictors that indicated the severity of the disease (*i.e*., bronchial stenosis and preoperative ECMO). However, adequate investigation, cardiopulmonary support, and treatment led to favorable outcomes.

## CONCLUSION

In conclusion, congenital tracheal stenosis is a complex disease requiring meticulous diagnostic strategies, optimal clinical compensation, and surgical treatment. Therefore, specific management should be performed in tertiary care centers with experienced professionals, cardiorespiratory support devices, and a multidisciplinary approach.
